# Defect Sites in Zeolites: Origin and Healing

**DOI:** 10.1002/advs.202104414

**Published:** 2021-11-27

**Authors:** Ana Palčić, Simona Moldovan, Hussein El Siblani, Aurelie Vicente, Valentin Valtchev

**Affiliations:** ^1^ Ruđer Bošković Institute Division of Materials Chemistry Laboratory for Synthesis of New Materials Bijenička cesta 54 Zagreb 10000 Croatia; ^2^ Institut des Sciences Appliquées de Rouen Rouen University Groupe de Physique des Matériaux (GPM) avenue de l'Université, BP12, Saint‐Étienne‐du‐Rouvray Cedex Rouen 76801 France; ^3^ Normandie Université ENSICAEN UNICAEN CNRS Laboratoire Catalyse et Spectrochimie 6 Boulevard Maréchal Juin Caen 14050 France; ^4^ The ZeoMat Group Qingdao Institute of Bioenergy and Bioprocess Technology CAS Laoshan District Qingdao CN‐266101 China

**Keywords:** Al/Si incorporation, defect sites, postsynthesis treatment, silanols, zeolites

## Abstract

This paper deals with the synthesis conditions–defect formation relationship in zeolites. Silicalite‐1 (MFI‐type) is used as a model material. Samples synthesized from a system with high basicity (at 100 °C), a system with moderate basicity (at 150 °C), and a fluoride‐containing system in neutral medium (at 170 °C) are compared. Well‐crystallized materials with sizes ≈0.1, 1–10, and 30–40 µm are obtained. The samples are analyzed by complementary methods providing information on the short‐ and long‐range order in the zeolite framework. A strong correlation between the number of point defects in the zeolite framework and preparation conditions is established. Silicalite‐1 synthesized under mild synthesis conditions from a highly basic system exhibits a larger number of framework defects and thus low hydrophobicity. Further, the calcined samples are subjected to aluminum and silicon incorporation by postsynthesis treatment. The Al/Si incorporation in the zeolite framework and its impact on the physicochemical properties is studied by XRD, TEM/SEM, solid‐state NMR, FTIR, and thermogravimetric analyses. The defects healing as a function of the number of point defects in the initial material and zeolite crystal size is evaluated. The results of this study will serve for fine‐tuning zeolite properties by in situ and postsynthesis methods.

## Graphical abstract

A quantitative correlation between zeolite synthesis conditions and defect formation is exemplified by studying MFI‐type materials. Postsynthesis modification with Si/Al results in Si/Al incorporation in the zeolite framework and healing point defects. The efficiency of the process in terms of the amount of healed defects with respect to their number in the parent material and zeolite crystal size is assessed.

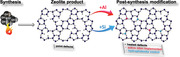

## 1 Introduction

The most commonly utilized molecular sieve materials at large scales are the microporous alumosilicate zeolites. These crystalline solids possess an ordered system of voids and/or channels.^[^ ^1^ ^]^ Their structure consists of tetrahedra involving a central T atom (T = Si, Al, Ge, B, etc.) surrounded by oxygen atoms at vertices. This basic element is further connected to the adjacent tetrahedron via shared oxygens.

The impact of zeolites on modern society in terms of the advancement of chemical process industries and environmental protection is immense. The zeolites represent the backbone of oil refining catalysts and petrochemicals production. They are used as catalysts in gas exhaust systems, as sorbents in gas separation, as water softeners in solid detergents, remediation of municipal water, capturing radioactive nuclides, etc.^[^ ^2^, ^3^, ^4^, ^5^, ^6^, ^7^, ^8^, ^9^ ^]^ The zeolite applications stem from their unique properties (shape‐selectivity, ion‐exchange capability, acidity/basicity, hydrophobicity/hydrophilicity, adsorption capacity) that are governed by their crystal structure (framework type) and chemical composition.^[^ ^10^ ^]^ Further, the size and morphology of the zeolite crystals substantially affect their performance in these applications.^[^ ^11^ ^]^ These zeolite features determine the effectiveness of the ion‐exchange process, impose diffusion limitations, control the quantity of adsorbed molecules as well as influence the (hydro)thermal stability of zeolite materials since the contact surface between the zeolite and the entity of interest depends on the size and shape of the crystal. The optimal performance of zeolite material results from a subtle interplay between their crystalline network, chemical composition‐related properties combined with the crystals' size and morphology. For this reason, it is necessary to bear in mind all the properties of zeolite crystals when considering their potential usage and related operations, e.g., their transport through pipes in industrial facilities.^[^ ^12^ ^]^ Accordingly, it is expected that processes of modulating the properties of zeolites via various top‐down approaches such as postsynthesis treatments will depend on the intrinsic properties of the crystal. For instance, the hollow structures harvested upon desilication of beta zeolite crystals retain the high crystallinity of parent material.^[^ ^13^ ^]^ In terms of postsynthesis fine‐tuning zeolite properties aiming to adjust the Si/Al ratio, the method proposed by Breck and Skeels was found to be particularly efficient for the enrichment of silicon content.^[^ ^14^ ^]^ Therein, FAU‐type material treated with ammonium hexafluorosilicate solution presented fewer framework hydroxyl vacancies and higher structural stability. Additionally, the amount of extra‐framework Al was reduced.

The preparation conditions determine the zeolite properties.^[^ ^10^ ^]^ Indeed, zeolite synthesis conditions directly affect the properties of the final product at all levels, from the nature of the T atom incorporated in the framework, the morphology, and the size of formed crystals, but also the level of crystallinity, the size of the coherent crystalline domains and the framework defects.^[^ ^15^, ^16^ ^]^ As an illustration, recently, Tanigawa et al. have shown that the variation in the Si/Al ratio in the synthesis mixture as well as changing the Si and/or Al sources yields CHA‐type materials of different sizes.^[^ ^17^ ^]^ Shinno et al. reported that the initial content of Ge controls the phase composition of the end product in the preparation of chiral STW‐type material.^[^ ^18^ ^]^ Besides, the addition of Mo to the preparation system resulted in nanosized Mo‐ZSM‐5 zeolite with higher structural stability under hydrothermal steaming conditions and fewer silanols, especially silanol nests defects, compared to the reference Mo‐free counterpart material.^[^ ^19^ ^]^ Clearly, these characteristics straightly determine the physicochemical properties of a zeolite, including the (hydro)thermal stability and the catalytic and adsorption performance. It was shown that the zeolite materials obtained from fluoride‐containing reaction media normally present larger crystals possessing fewer defects rendering them less prone towards deactivation and consequently more active as catalysts.^[^ ^20^, ^21^ ^]^ The defect sites were also identified as one of the factors affecting the hydrothermal stability of zeolites, thus representing a bottle‐neck for their application since they exhibit a higher affinity towards water.^[^ ^21^ ^]^ Point defects in zeolite structure, i.e., a missing T atom within zeolite lattice, are manifested as silanol nests—a group of four Si‐OH groups which may interact via hydrogen bonds.^[^ ^22^ ^]^ Other common types of defects in zeolites are associated with nonbridging oxygen atoms (≡Si–O^−^), which occur through incomplete condensation of silicate species during zeolite synthesis.^[^ ^23^ ^]^

This study is dedicated to the synthesis–properties relationship in zeolites by establishing the dependence of zeolite features on the preparation conditions. Particularly, the focus of this study is the impact of physical and chemical parameters on zeolite framework defects formation. Besides, the study demonstrates how the drawback of a large number of structural defects could be turned into an advantage that facilitates the engineering of zeolite properties by postsynthesis treatment.

## 2 Results and Discussion

### 2.1 Characterization of the Silicalite‐1 Materials

**Figure 1:**
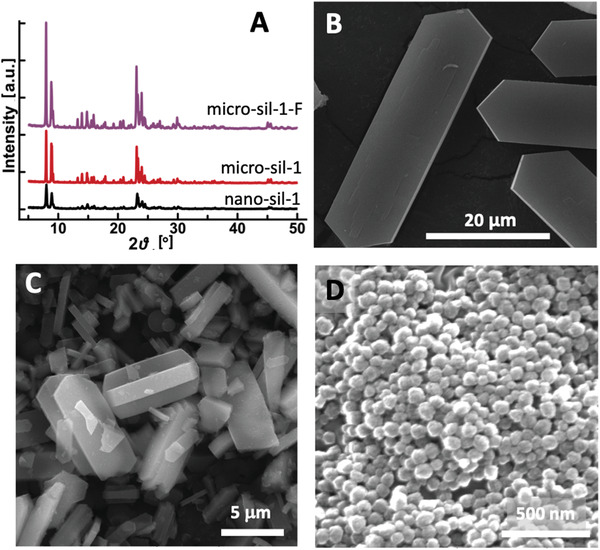
XRD patterns A) of the studied silicalite‐1 materials and their respective SEM images: B) micro‐sil‐1‐F, C) micro‐sil‐1, D) nano‐sil‐1.

Powder X‐ray diffraction (XRD) patterns (**Figure** **1**) of the studied silicalite‐1 samples present the reflections corresponding to the MFI‐type zeolite framework. Considering that the XRD measurements were conducted under identical conditions, the highest peak intensities and the cumulative pattern intensity in the micro‐sil‐1 samples signify a higher ordering degree. The nanosized silicalite‐1 crystals are uniform in size and morphology, as shown in the scannin electron microscopy (SEM) image in Figure 1. According to the dynamic light scattering (DLS) analysis (Figure S1, Supporting Information), the nano‐sil‐1 ranges from 60 to 230 nm, with a maximum at 114 nm. The crystals are rounded without any expressed crystal face. In contrast, the coffin‐shaped morphology with well‐developed faces was observed in micrometer‐sized MFI samples (Figure 1). Such features are common for MFI‐type materials. The size of the crystals obtained from OH^−^ and F^−^ medium is 1–10 and 30–40 µm, respectively.

The morphological features of the three samples reflect in the N_2_ physisorption isotherms (Figure S2, Supporting Information). The nanosized MFI‐type materials exhibit a combination of type I and type IVa physisorption isotherm with H4 hysteresis loop at *p*/*p*_0_ >0.8 typical for aggregated nanosized particles.^[^ ^1^ ^]^ On the other hand, micrometer‐sized MFI‐type materials exhibit type Ia isotherm with a sharp uptake at low relative pressures followed by horizontal adsorption and desorption branches, indicating a relatively small external surface area.^[^ ^1^ ^]^ All materials present high micropore volume in accordance with their high crystallinity (**Table** **1**). Besides, the *S*_BET_ area and *V*_meso_ value of the nanosized sample (501 m^2^ g^−1^, 0.43 cm^3^ g^−1^) are higher than those of the micrometer‐sized ones (371 m^2^ g^−1^, 0.01 cm^3^ g^−1^; 375 m^2^ g^−1^, 0.02 cm^3^ g^−1^) due to the presence of textural mesopores between the zeolite crystals.

**Table 1:** XRD, nitrogen physisorption, chemical, TG, NMR, and IR analyses of the studied series of MFI‐type zeolite materials

Sample	*f*_C_ ^a)^	*S*_BET_ [m^2^ g^−1^]	*V*_mic_ / [cm^3^ g^−1^]	*V*_meso_ [cm^3^ g^−1^]	Si/Al^ICP^	SiOH_ext_ [μmol_H_ g^−1^]	SiOH_int_ [μmol_H_ g^−1^]	weight loss [%]^b)^	*Δz* [μmol_H_ g^−1^]^c)^
nano‐sil‐1	1	501	0.16	0.43	–	1083	2084	7.0 (9.6)	0
nano‐sil‐1‐Al	1.06	518	0.16	0.41	45	1901	1742	7.4 (9.6)	−17
nano‐sil‐1‐Si	1.09	387	0.15	0.30	–	703	706	1.4 (1.8)	−1947
micro‐sil‐1	1	371	0.17	0.01	–	140	744	1.7 (2.6)	0
micro‐sil‐1‐Al	1.15	385	0.17	0.03	73	102	575	2.7 (4.2)	−157
micro‐sil‐1‐Si	0.92	427	0.17	0.03	–	139	546	1.2 (1.7)	−202
micro‐sil‐1‐F	1	375	0.18	0.02	–	25		0.6 (0.8)	–
nano‐ZSM‐5	1	516	0.17	0.38	42	2478	2877	8.7 (11.5)	0
nano‐ZSM‐5‐Al	1.04	448	0.14	0.37	14	3169	1115	11.5 (15.3)	−716
nano‐ZSM‐5‐Si	1.14	350	0.15	0.20	57	786	2420	8.5 (11.1)	−1885

^a)^ *f*_C_ – relative crystallinity calculated with respect to parent material in each series;

^b)^ weight loss up to 200 °C and for the whole measurement range (25–800 °C; values in the brackets);

^c)^ defect structure factor *Δz* is calculated according to the previously published method using nano‐sil‐1 as a reference material.^[^ ^14^ ^]^

**Figure 2:**
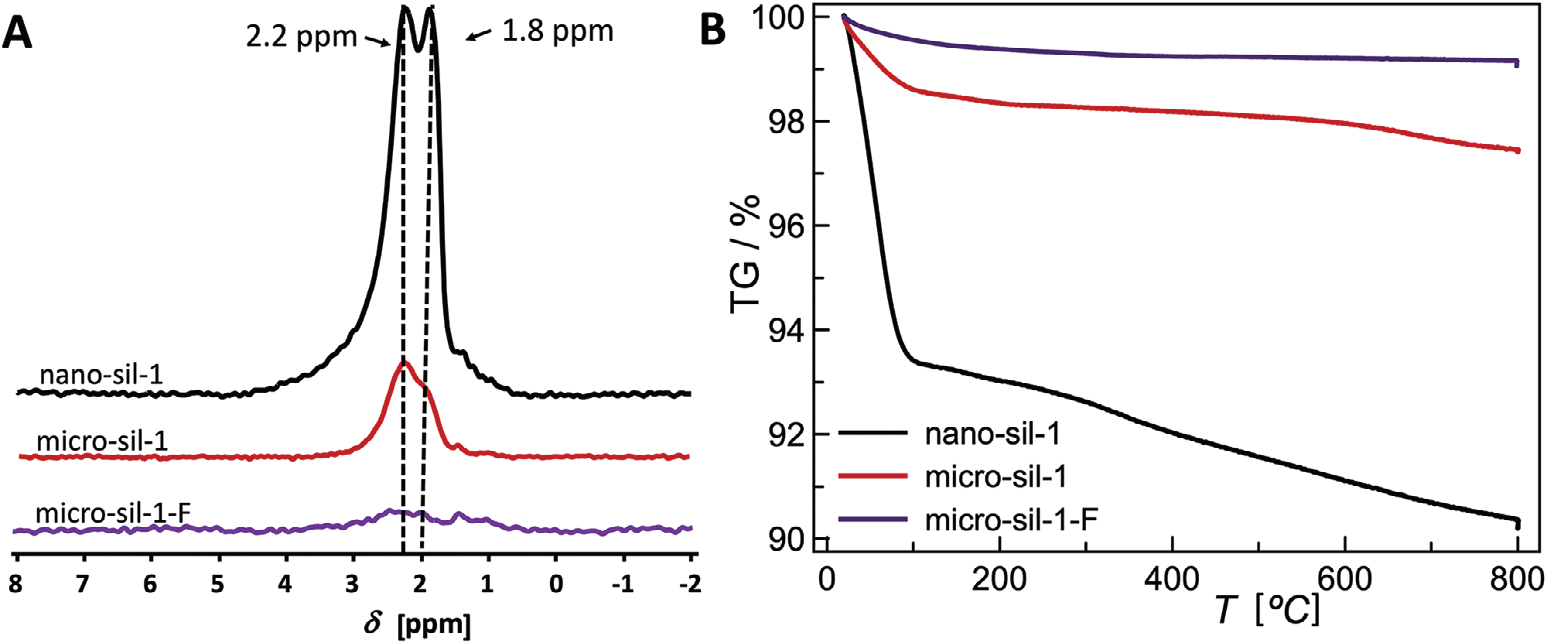
^1^H MAS NMR spectra of silicalite‐1 samples prepared from different synthesis mixtures A) dehydrated at 400 °C in vacuum line and B) TGA curves of the silicalite‐1 samples upon hydration in 77% humidity atmosphere.

Silanol groups in zeolites develop on sites where framework bonds are terminated, such as on the outer surface of the crystal or at the framework defects. Herein, different silanols, representative of different types of defects, were studied by ^1^H magic‐angle spinning nuclear magnetic resonance spectroscopy (MAS NMR) (**Figure** **2**A), and their respective amounts are summarized in the Table 1 (column *Δz*). The set of experimental results reveals a strong impact of synthesis conditions on the number of silanols. In line with previous findings, F‐medium yields nearly defect‐free crystals, whereas, in the OH‐system, the samples present a significant amount of SiOH groups. Moreover, fewer defects are generated at higher synthesis temperatures, i.e., 150 and 170 °C. The use of a highly basic TPA‐rich mixture at low crystallization temperature causes generation of the largest amount of framework defect in nanosized crystals. The number of framework defects in micrometer‐sized crystals is lower, which is a consequence of lower basicity and higher crystallization temperature. Namely, in the OH^−^ systems for the synthesis of all‐silica zeolites the positive charge of the structure‐directing agent is balanced by siloxy defects, ≡Si–O^−^. Thus, increased concentration of hydroxide anions, therewith increased concentration of cations, contributes to the generation of a higher number of framework defects as more silanols get deprotonated to compensate the charge of cations. Further, at elevated reaction temperatures the coalescence and ordering of silicate species occur more rapidly, meaning that the crystal growth rate is faster under these conditions. However, the supersaturation is lower at higher temperatures due to increased solubility. Generally, the dominant crystal growth mechanism at low supersaturations is spiral growth resulting in crystals of a smooth surface. At higher supersaturation values, prevails 2D growth mechanism via adhering growth units to the surface nuclei. Statistically, stacking sequences discordance becomes more probable when there are more nutrients. Hence, defect‐rich crystals of rough surface are obtained from highly supersaturated systems. Consequently, when the reaction temperature rises and supersaturation becomes lower, the organization of the zeolite building units is more precise and less prone to the formation of defect sites. Herein, the lowest number of framework defects is detected in the fluoride medium synthesized sample due to the slow crystal growth rate (the supersaturation in fluoride media is lower) as well as the ability of the fluoride anion to balance the positive charge of TPA^+^ cation in the zeolite structure by forming pentacoordinated [(Si–O–)_4_SiF^−^]. The subtle interplay of described phenomena, leads to the crystallization of materials of different properties. These conclusions are based on the combined ^1^H NMR thermogravimetric (TG) analysis (Figure 2B). The presence of silanols in silicalite‐1 renders the materials partially hydrophilic, which was demonstrated by ^1^H NMR spectra of a studied set of all‐silica MFI‐type zeolites upon water adsorption and by the TG analysis of hydrated samples (Figure 2B, Table 1; Figure S2B, Supporting Information). Namely, the amount of physisorbed water on zeolite materials (associated with weight loss up to 200 °C; Table 1, values in the brackets) is higher as the number of SiOH defects in zeolite crystals gets augmented as substantiated by water adsorption experiments.^[^ ^23^ ^]^

To sum up, we prepared highly crystalline MFI‐type zeolite materials with different morphology, size of the crystals, and hydrophobicity/hydrophilicity. Nanosized silicalite‐1 (60–230 nm) with relatively high hydrophilicity was prepared in a highly alkaline system at low crystallization temperature (100 °C). The silicalite‐1 crystals synthesized at higher temperature (150 °C) at moderate alkalinity exhibit certain hydrophilicity since they adsorb 2.6 wt% water in a 77% humidity atmosphere. On the contrary, crystallization in a neutral fluoride‐containing system at high temperature (170 °C), where slow crystal growth is favored, affords large, almost defect‐free, MFI crystals (30–40 µm) of low affinity towards water (0.84 wt%). Specifically, at higher OH^−^ concentration, the ratio ≡SiO^−^/≡SiOH increases and prevents the complete condensation to ≡Si–O–Si≡; this effect is accentuated when a relatively low synthesis temperature is used. In the F‐medium, the pH is lower, and there are fewer ≡SiO^−^ entities. Besides, the slow crystal growth limits defect formation. Further, the zeolites having a high amount of defects were subjected to postsynthesis treatment with solutions of Al or Si compounds aiming to incorporate additional framework atoms and modify/improve their physicochemical properties. For the sake of comparison, nanosized ZSM‐5 samples (90–400 nm) were also included in this set of experiments.

### 2.2 Postsynthesis Modification of MFI‐Type Zeolite

XRD patterns of Al and Si modified samples (Figure S3, Supporting Information) show high and even improved crystallinity in respect to the parent materials (Table 1). Moreover, the particle size remains unaffected by any applied modification procedures as revealed by SEM and DLS analyses (Figures S4 and S5, Supporting Information). Nitrogen adsorption (Table 1; Figure S6, Supporting Information) shows minor fluctuations of micropore volume in studied zeolites. In contrast, external surface area and mesoporous volumes indicate that any moieties get deposed on the crystal surface of crystals during the treatment.

**Figure 3:**
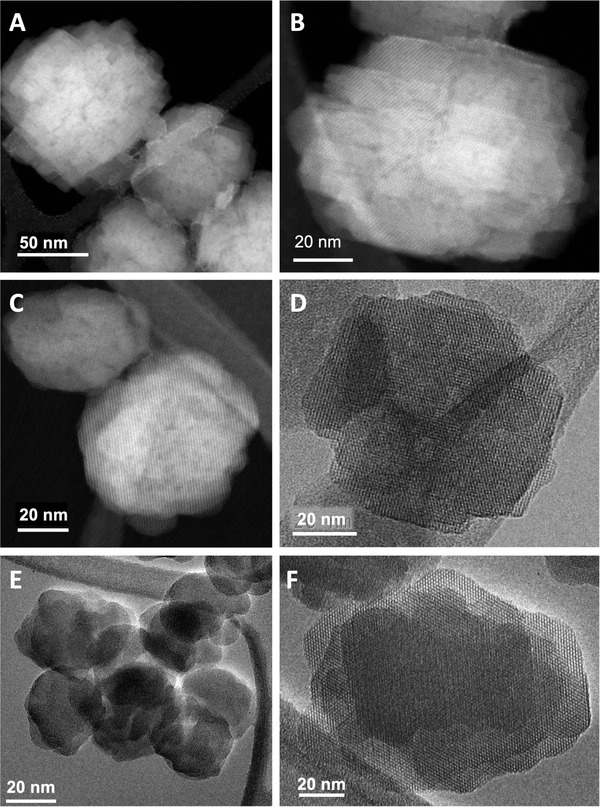
High resolution TEM images of A,B) nano‐sil‐1, C,D) nano‐sil‐1‐Al, and E,F) nano‐sil‐1‐Si.

Transmission electron microscopy (TEM) analysis of the series of nanosized silicalite‐1 samples (**Figure** **3**) reveals that the respective nanoparticles are complex agglomerates constituted by the superposition of thin MFI crystallites. In line with DLS findings, the crystals’ size is constant in all of the studied samples. Moreover, the crystal morphology remains preserved in the treated samples. Further, no amorphous layer is observed on the surface of the particles after the postsynthesis treatment. Finally, the scanning transmission electron microscopy energy‐dispersive X‐ray spectroscopy (STEM EDX) analysis (Figure S7, Supporting Information) shows that in the aluminated nano‐sil‐1 the Al is uniformly distributed within the crystals.

The set of experimental results show that the postsynthesis Al and Si incorporation in MFI framework does not affect the zeolite intrinsic features in terms of their crystallinity, textural properties, size, and morphology. However, to evaluate the potential impact of postsynthesis framework modification on the performance of newly obtained materials, atomic‐level information is required. Therefore, a comprehensive spectroscopic investigation has been conducted to ascertain the features of modified materials from the point of view of their water adsorption affinity, the nature of the existent silanol groups, including the Al and Si atoms' local environment. The collected results are presented in the following sections.

#### 2.2.1 Thermogravimetric Analysis

The weight loss observed in the TG and respective differential (dTG) curves of the hydrated studied MFI‐type materials (Table 1; Figure S8, Supporting Information) is ascribed to the removal of water molecules since the main events in all samples occur below 200 °C. As expected, the total weight loss is higher in the nanosized samples having greater specific surface area and more defects than the micrometer‐sized materials. Compared to the initial samples, in the Si‐treated materials, the general trend is less water adsorbed, whereas the aluminated materials present a larger or identical amount of adsorbed water. Notably, despite the distinct quantity of the adsorbed water on the initial silicalite‐1 materials, the water amount is nearly equal in the Si‐treated samples, no matter the crystal size. The results indicate that the silication renders the zeolites more hydrophobic, while the Al‐treatment increases the affinity towards water. Moreover, in term of reducing the water adsorption capacity, the silication causes a more pronounced effect in the nanosized materials.

#### 2.2.2 IR Analysis

**Figure 4:**
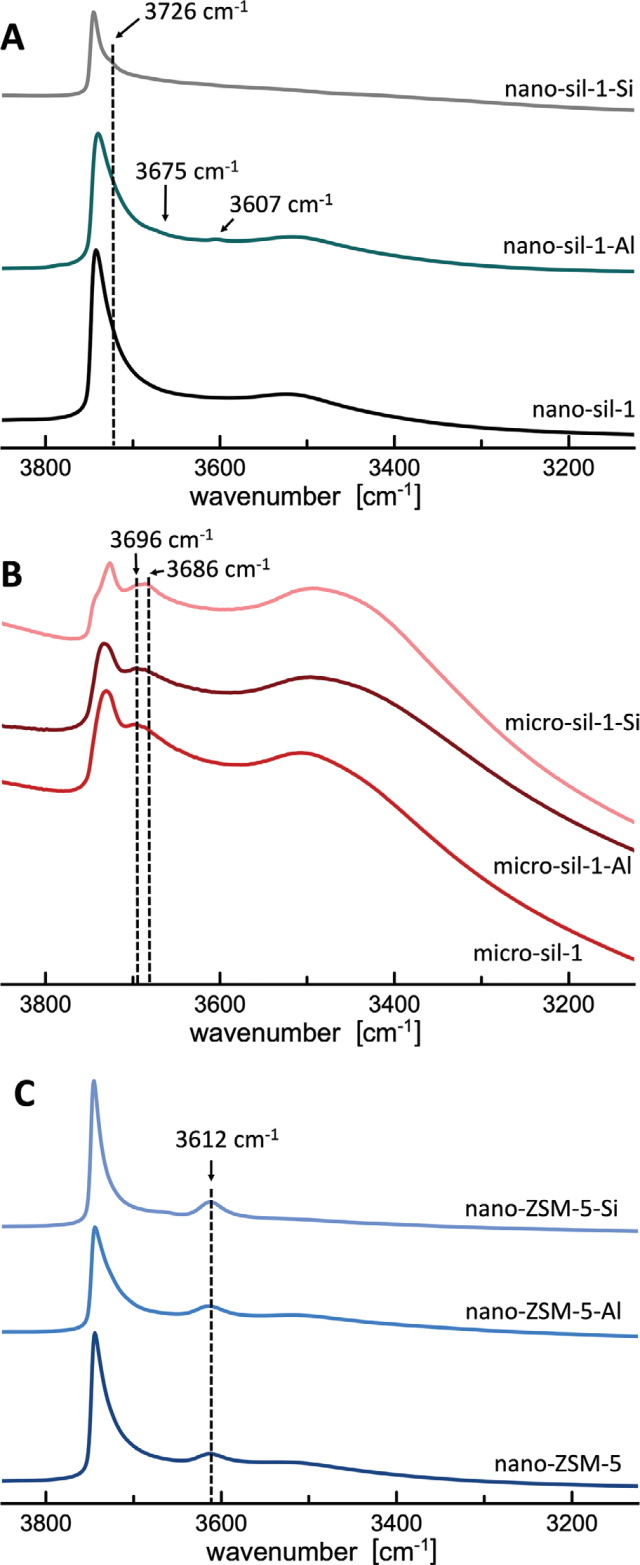
Normalized IR spectra in the hydroxyl groups region of the studied MFI‐type zeolite materials (A) nano‐sil‐1; B) micro‐sil‐1; C) nano‐ZSM‐5).

The differences in the infrared (IR) spectra in the hydroxyl groups region (**Figure** **4**) of the studied samples are reflected as the net change in defect structure factors between the starting zeolite and the product material (*Δz*, Table 1). It enables the estimation of the relative number of defect sites as reported elsewhere.^[^ ^14^ ^]^ Herein, the total amount of silanols determined on the grounds of ^1^H NMR of the parent material was taken as the reference value. The declining defect structure factors of the modified samples strongly imply that both kinds of processes render defect healing. From that point of view, the silication is more efficient than alumination. Besides, the smaller size, i.e., larger external surface available for contact with the treating agent and shortened diffusion path‐way, causes a more expressed effect. In addition, for micro‐sil‐1 the outcome is less dependent on the nature of the modification process. Furthermore, the collected spectra provide insight into the Brønsted acid sites in the studied materials. In ZSM‐5 series (band at 3612 cm^−1^) the Brønsted sites remain unaltered in both Al‐ and Si‐treated samples, while in nano‐sil‐1‐Al some are generated (3607 cm^−1^). This redshift of the Brønsted acid sites band compared to ZSM‐5 is ascribed to the perturbation of the bridging OH groups by extra‐framework Al (EFAl) species rendering these entities highly acidic.^[^ ^24^ ^]^ Moreover, in nano‐sil‐1‐Al the EFAl is indicated by the band at 3675 cm^−1^.^[^ ^25^ ^]^ Finally, it is interesting that no matter the treatment, the silanol nests (3550–3400 cm^−1^) remain in the micrometer‐sized series presenting a redshift of the bands’ maximum, thus indicating a difference in the H‐bonds among the SiOH groups.^[^ ^26^ ^]^ Besides, in the micrometer‐sized series, are observed bands at 3696 and 3686 cm^−1^ that are assigned to loosely H‐bonded hydroxyl groups (almost free) located at internal positions.^[^ ^27^ ^]^

#### 2.2.3 NMR Study

**Figure 5:**
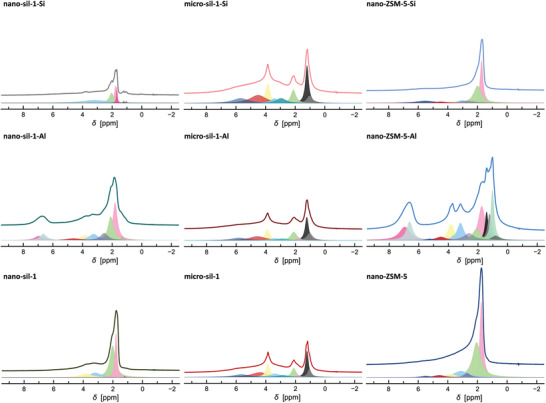
Normalized ^1^H MAS NMR spectra of studied MFI‐type zeolite materials with the respective deconvolution curves. The value of *y*‐axis is identical in all spectra.

The deconvoluted ^1^H NMR spectra of the studied materials are depicted in the **Figure** **5** and the respective peak assignations are given in the Table S1 in the Supporting Information. The spectra reveal a certain amount of retained adsorbed water despite the heating at 200 °C preceding the experiment. Again, there is a marked difference between micrometer and nanosized samples subjected to postsynthesis treatment. As to the initial material, the profile of the micrometer‐sized samples' spectra is rather unaltered upon the modification. Still, the difference is the enlarged contribution of silanol nests (4.5 ppm) in the spectrum of micro‐sil‐1‐Si and thus a higher quantity of water (2.8, 5.7 ppm). Further, the postsynthesis process changes the distribution of H species in the nanosized MFI‐type materials. Besides, depending on the method, some moieties become more discerned and/or new ones appear. The Al incorporation leads to the generation of Brønsted sites and EFAl species in nano‐sil‐1 (4.4, 7, 2.5 ppm).

Furthermore, in this material, the features of isolated silanol groups (1.05, 1.2 ppm) become more expressed. Analogously, the resonances corresponding to bridging SiOHAl groups (4.5, 5.5 ppm) and EFAl (2.7 ppm) get enhanced in nano‐ZSM‐5‐Al, confirming the incorporation of supplementary Al atoms in the framework. The alumination, however, generates additional aluminous deposits within the zeolite voids. Besides, the emergence of the bands at 0.8 and 7 ppm suggests the formation of new types of EFAl groups and Brønsted acid sites, respectively. Also, other nonhydrogen bonded silanols develop, giving rise to peaks at 1.05, 1.2, and 1.4 ppm. In both Al‐treated nanosized zeolites, a signal at 6.7 ppm that stems from the NH_4_^+^ charge‐balancing cations is observed. The presence of ammonium is a consequence of the use of NH_3_ solution with the aim to extract the excess Al. The outcome of the treatment of nanosized MFI‐type materials with (NH_4_)_2_[SiF_6_] is the diminution of the total spectral intensity compared to the initial materials. Regarding the number of H atoms in the studied samples, the results indicate the reduction of silanols, which means that the net amount of defect sites in the zeolite framework has declined. Likewise, less silanols signify fewer sites available for bonding/adsorbing water. Further, in Si‐modified nanosized ZSM‐5 are present tetrahedral Al sites along with the enlarged fraction of the extra‐framework species (2.7 ppm). Namely, the relative contribution of this signal to the total spectral intensity is 5.5% in nano‐ZSM‐5, whereas in nano‐ZSM‐5‐Si it reaches 8%. Hence, it is deduced that the framework Al is partially eliminated during the silication process.

**Figure 6:**
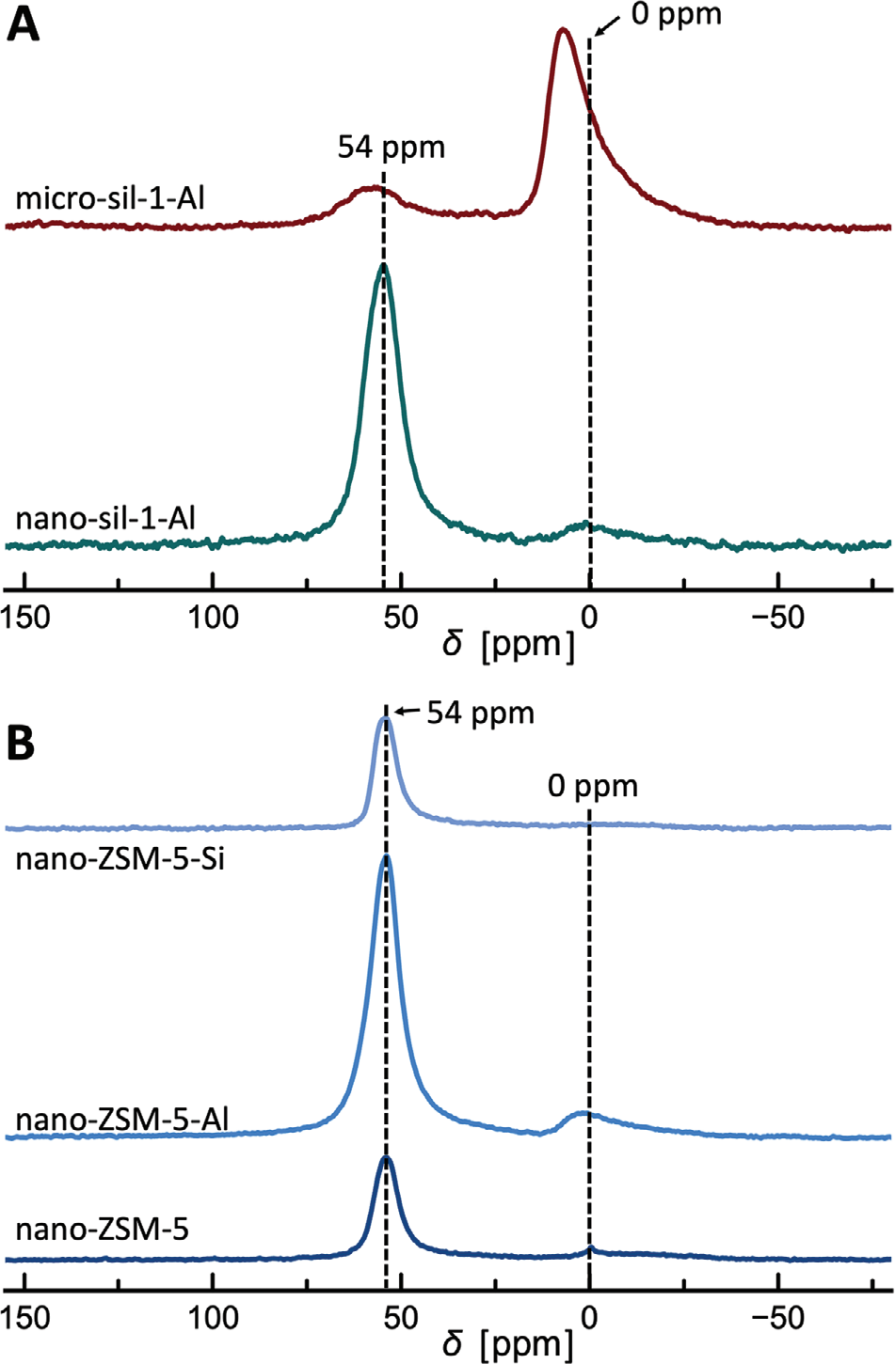
Normalized ^27^Al MAS NMR spectra of A) aluminated silicalite‐1 samples and B) a series of nanosized ZSM‐5 samples.

^27^Al NMR spectra of the studied MFI‐type samples (**Figure** **6**) provide information on the Al environment in these materials. Postsynthesis modification with AlCl_3_ solution induces incorporation of Al into zeolite framework (tetrahedral coordination, 54 ppm; 3607 cm^−1^ in IR; 4.5 ppm in ^1^H NMR) and formation of a small fraction of extra‐framework octahedral Al species in nano‐sil‐1‐Al (0 ppm; 3675 cm^−1^ in IR; 2.7 ppm in ^1^H NMR). On the other hand, in micro‐sil‐1‐Al the dominant Al entities are extra‐framework octahedrally coordinated dimers and/or trimers found at 6.5 ppm with some tetrahedral Al having a somewhat different environment than in nano‐sil‐1‐Al and ZSM‐5 since the corresponding peak lies at 58 ppm.^[^ ^28^ ^]^ Furthermore, higher intensity of 54 ppm signal indicates that in zeolite ZSM‐5, additional Al atoms are embedded into the framework upon the alumination (3612 cm^−1^ in IR; 4.5, 5.5 and 7 ppm in ^1^H NMR). Besides, via this approach are produced octahedral EFAl moieties giving rise to the resonance at 1.5 ppm. Potentially, some of these species may be correlated with the 0.8 ppm signal observed in ^1^H NMR spectrum. A slight decrease of the area of the resonance around 0 ppm in the nano‐ZSM‐5‐Si‐calc compared to the initial material indicates that some extra‐framework Al deposits in zeolite voids may be extracted during the treatment with (NH_4_)_2_[SiF_6_].^[^ ^29^ ^]^

**Figure 7:**
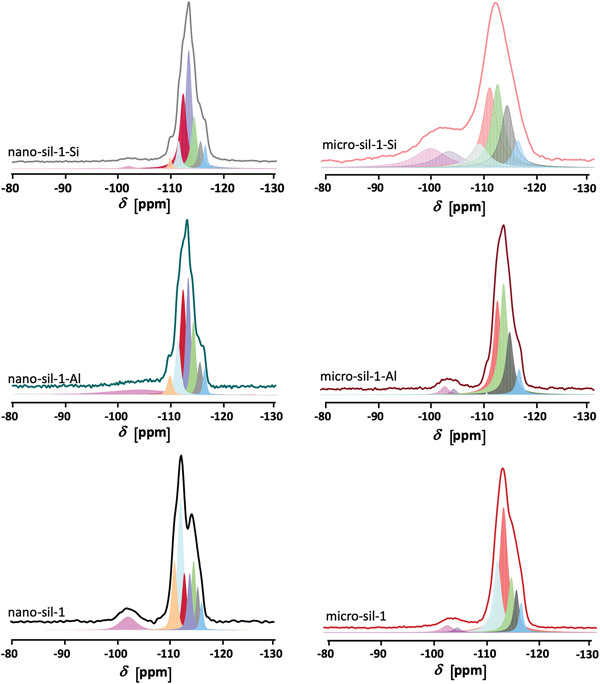
^29^Si MAS NMR spectra and the respective deconvolution curves of the parent silicalite‐1 samples and the Al‐ and Si‐treated counterparts.

The ^29^Si NMR spectra (**Figure** **7**) of the initial silicalite‐1 samples are not well resolved, suggesting inhomogeneity of environments of Si atoms and thus the presence of structural defects. These spectra are decomposed into several signals that represent at least 12 nonequivalent crystallographic sites (T sites) in the MFI‐type materials. The contribution of the peaks at −91 ppm that originate from geminal Q^2^ ([(HO)_2_‐Si‐[(OSi)_2_]) Si species is almost negligible. The signals at −102 ppm correspond to Q^3^ ([(HO)‐Si‐[(OSi)_3_]) silicon sites and become amplified in the ^29^Si {1H} cross‐polarized (CP) NMR spectrum (Figure S9, Supporting Information). Close inspection of the micro‐sil‐1 spectrum reveals two different environments of Q^3^ silicon species manifested by two components contributing to the shoulder at −102 ppm. This is correlated with the two types of silanol groups found in the IR spectra: free (3755–3700 cm^−1^) and loosely H‐bonded OH groups (3696 and 3686 cm^−1^). Q^4^ ([Si‐[(OSi)_4_]) features exhibit signals between −108 and −118 ppm. The postsynthesis treatments result in more discerned ^29^Si NMR spectra in the case of both nano‐sil‐1‐Al and nano‐sil‐1‐Si. Besides, the relative area of the Q^3^ signal diminishes and is particularly low in nano‐sil‐1‐Si. Furthermore, in the respective ^29^Si {^1^H} CP NMR spectrum, the band of Q^3^ Si is the most prominent while the −91 and −114 ppm signals are fairly enhanced. Although this kind of spectrum is not quantitative, the observation that the ^29^Si {^1^H} CP NMR spectrum of the nano‐sil‐1‐Si sample is rather poor compared to the initial material (higher signal‐to‐noise ratio in nano‐sil‐1) implies that the total amount of defects is lower in the samples treated with (NH_4_)_2_[SiF_6_].

Hence, it is deduced that the incorporation of T atoms in the zeolite framework by this mode of postsynthesis treatment is highly efficient in terms of defects’ healing. Opposite to the effect in nano‐sil‐1‐Si, silication of micrometer‐sized material decreases the ordering degree reflected in less resolved ^29^Si NMR spectrum of micro‐sil‐1‐Si than of the initial material. Besides, the fraction of Q^3^ silicon sites is amplified in the treated sample. However, in ^29^Si {1H} CP NMR spectrum of micro‐sil‐1‐Si, the relative contribution of Q^4^ is higher than the Q^3^ species. Likewise, the overall intensity of the ^29^Si {^1^H} CP NMR spectrum of the micro‐sil‐1‐Si is higher than for the parent sample. Clearly, the silication of micrometer‐sized silicalite‐1 is not as effective as in nanosized material, and additional defect sites were generated via this process. This could be explained by fundamental properties of nano‐ and micrometer‐sized crystals—larger crystals have lower external surface area exposed to contact with the solution of silication agent. Moreover, nano‐sil‐1 is a hierarchical material presenting mesopores smaller than 5 nm (TEM images, Figure 3), which also prompts achieving proximity of silication agent and crystal surface. Hence, with respect to nanosized crystals the micro‐sized silicalite‐1 is less adapted to postsynthesis modifications. Thus, the efficiency of postsynthesis modification on micrometer‐sized crystals is lower. Besides, not each incorporation of a new framework atom is completely successful, i.e., a discontinuity between SiO_4_ tetrahedra (Q^3^ site) might occur. Recent findings on effective defect healing via recrystallization in water solution of TEAOH and NH_4_F at 170 °C suggest that further optimizing the conditions of the silication treatment (temperature, duration period, concentration of ammonium hexafluorosilcate) should produce materials with higher connectivity levels.^[^ ^30^ ^]^

## 3 Conclusion

We studied the number of framework defects in silicalite‐1 crystals as a function of synthesis conditions. It was found that the basicity of the system and the crystallization temperature have a pronounced effect on the number of framework defects and, accordingly, hydrophobicity/hydrophilicity of the zeolite. Thus, the material synthesized at a relatively low temperature (100 °C) from a highly basic system exhibited a larger number of framework defects, while the one synthesized from a neutral system at a high temperature (170 °C) presented the lowest quantity of silanol‐type defects. Hence, the framework defect number in a zeolite is determined by the complex interplay between the synthesis medium, the reaction temperature, and the crystal growth rate. Namely, the nucleation and crystal growth rate are a function of saturation of silicate species that is low in fluoride medium rendering slow crystal growth and the formation of almost defect‐free crystals. In contrast, the concentration of OH^−^ ions governs the condensation as well as protonation of silicate anions. This effect is stronger at lower temperatures leading to a less‐connected framework.

We developed procedures for postsynthesis Al and Si inserting in the zeolite framework. Upon being subjected to these procedures, zeolites possessing a higher number of framework defects have produced materials with fewer defect sites. The T atom anchoring into lattice positions and the subsiding of silanol defects is particularly noticeable in the case of nanosized MFI‐type materials. Indeed, it is surmised that the larger external surface and shortened diffusion path of nanocrystals contribute to the efficiency of the postsynthesis modification process. Further, no matter the sample, more silanol defects are healed in the presence of a silication agent than due to Al treatment.

The collected set of results delivered findings on the opportunities of engineering defect sites within zeolite crystals via a direct synthesis approach as well as by employing postsynthesis modification processes. Guidelines to prepare materials possessing fewer point defects and consequently of higher hydrophobicity are provided and represent the basis for designing zeolite materials of enhanced (hydro)thermal stability.

## 4 Experimental Section

### Zeolite Preparation

The nanosized silicalite‐1 zeolite (sample denoted as nano‐sil‐1) was synthesized using tetrapropylammonium hydroxide (TPAOH, Alfa Aesar, 1 m), tetraethoxysilane (TEOS, Aldrich, 98%), and doubly distilled water produced in laboratory. First, the needed amount of TPAOH was mixed with the water in a polypropylene bottle, followed by the addition of TEOS, yielding a reaction mixture with the following molar composition 25 SiO_2_: 9 TPAOH: 480 H_2_O. TEOS was hydrolyzed overnight and subsequently hydrothermally treated at 100 °C for 30 h in a preheated convection oven. The recovered solid phase was washed with distilled water until a neutral pH was attained. The washed material was calcined at 550 °C for 5 h. The temperature of 550 °C was reached with a heating rate of 1.75 °C min^−1^.

The micrometer‐sized silicalite‐1 zeolite (sample denoted as micro‐sil‐1) was obtained from the synthesis mixture having the molar oxide composition 25 SiO_2_: 3 TPAOH: 1500 H_2_O prepared by admixing the needed amounts of TPAOH, water, and TEOS in a polypropylene bottle. After the hydrolysis of TEOS that was conducted overnight, the reaction mixture was charged into Teflon‐lined autoclave and placed into a convection oven preheated at 150 °C for 24 h. The recovered solid phase was treated in the same way as the nano‐sil‐1 material, i.e., washed with water until a neutral pH and calcined at 550 °C for 5 h. The temperature of 550 °C was attained with a heating rate of 1.75 °C min^−1^.

Micrometer‐sized silicalite‐1 crystals were prepared using F^−^ as mineralizer (sample denoted as micro‐sil‐1‐F) from the system 1 SiO_2_: 0.08 TPABr: 0.04 NH_4_F: 20 H_2_O by mixing the appropriate amounts of water, fumed silica (SiO_2_, Sigma, 99.8%), tetrapropylammonium bromide (TPABr, 98%, Aldrich) and ammonium fluoride (NH_4_F, 98%, Aldrich). The synthesis was performed at 170 °C for 9 d. The recovered powder was washed with water until a neutral pH and calcined for 5 h at 550 °C. The temperature of 550 °C was attained with a heating rate of 1.75 °C min^−1^.

The nanosized ZSM‐5 zeolite (sample denoted as nano‐ZSM‐5) was prepared from the reaction system having the initial Si/Al = 150 and molar composition 25 SiO_2_: 0.0833 Al_2_O_3_: 9 TPAOH: 480 H_2_O. TPAOH, doubly distilled water, TEOS, and aluminum isopropoxide (Fluka, ≥98%) were stirred overnight in a polypropylene bottle and transferred into a preheated convection oven at 100 °C for 3 d. The final product was harvested by centrifugation and washing with water until reaching a neutral pH followed by calcination for 5 h at 550 °C. The temperature of 550 °C was reached with a heating rate of 1.75 °C min^−1^.

### Postsynthesis Modification

The alumination procedure was conducted by suspending 1 g of calcined MFI‐type zeolite material in 50 g 0.4 wt% aluminum chloride hexahydrate (Merck, 97%) solution. The mixture was stirred at 80 °C over‐night. After the treatment, the solution was decanted, and the excess Al was washed away first by washing with NH_3_ solution (*c*(NH_3_) = 0.1 mol dm^−3^, Alfa Aesar) and then with distilled water. The separation of the liquid and solid parts was made by centrifugation. The aluminated samples were denoted by adding the suffix “‐Al” to the label of the sample, e.g., “micro‐sil‐1‐Al” stands for aluminated micrometer‐sized silicalite‐1 sample.

The silication procedure was carried out at 80 °C for 72 h. Herein, 1 g of calcined MFI‐type zeolite material was dispersed in 54 g of water and agitated at 80 °C. After 1 h, when the temperature of the suspension was equilibrated, 25 g of an aqueous solution of ammonium hexafluorosilicate (*c*(NH_4_)_2_[SiF_6_]) = 1.6 × 10^−6^ mol dm^−3^, Prolabo, 98%) was added dropwise.^[^ ^14^ ^]^ The solid was recovered by centrifugation and repeatedly washed with hot distilled water. Finally, it was calcined at 550 °C for 5 h. The silicated samples were designated by adding the suffixes “‐Si” to the label of the sample, e.g., “micro‐sil‐1‐Si” represents micrometer‐sized silicalite‐1 sample that was treated with (NH_4_)_2_[SiF_6_] solution and subsequently calcined for 5 h at 550 °C (heating rate of 1.75 °C min^−1^).

### Physicochemical Characterization

The powder X‐ray diffraction of the samples was measured employing a PANalytical X'Pert Pro diffractometer with Cu *Kα* radiation (*λ* = 1.5418 Å, 45 kV, 40 mA). The electron images of the prepared crystals were collected by MIRA‐LMH (Tescan) SEM equipped with a field emission gun. For TEM analysis, an analytical double (objective and probe) corrected JEOL ARM200CF microscope equipped with a 100 mm Centurio EDS detector was employed. An accelerating voltage of 120 kV was applied. HR STEM‐HAADF (high resolution scanning transmission electron microscopy high‐angle annular dark‐field imaging) and BF TEM (bright‐field transmission electron microscopy imaging) approach were employed for the medium and high‐resolution imaging and STEM EDX for assessing the chemical composition. The camera length was fixed at 8 cm.

The textural properties of the samples were assessed on the grounds of the nitrogen adsorption/desorption isotherms using a Micrometrics 3Flex volumetric adsorption analyzer. Prior to the measurement, the samples were degassed at 300 °C under a vacuum overnight. The specific surface area, *S*_BET_, was calculated according to the Brunauer–Emmett–Teller method, whereas the total pore volume was taken from the nitrogen adsorbed volume at *p*/*p*_0_ = 0.95. The *t*‐plot method was employed for the estimation of the micropore volume. Further, the mesoporous volume was determined as the difference between the total and micropore volumes, *V*_meso_ = *V*_total_−*V*_mic_.

The solid‐state ^29^Si, ^1^H, and ^29^Si {^1^H} cross‐polarized (CP) magic‐angle spinning (MAS) NMR spectra were acquired on a Bruker Avance III‐HD 500 (11.7 T) spectrometer using 4 mm‐OD zirconia rotors. Single‐pulse excitation of 2.3 µs was used for ^29^Si MAS NMR experiment and 20 s of recycling delay at a spinning frequency of 12 kHz. ^1^H MAS NMR was performed on samples dehydrated at 200 °C. A Hartman–Hann echo was used with a *π*/2 pulse of 4.25 µs, a spinning rate of 14 kHz, and a recycle delay of 2 s. ^29^Si {^1^H} cross‐polarized (CP) MAS NMR spectra were recorded with a contact time of 7.5 ms and a recycling time of 3 s. Finally, ^27^Al MAS NMR was performed on the Bruker Avance 400 spectrometer with a spinning speed of 14 kHz, pulse of 0.9 µs and recycle delay of 1 s. TMS was used as a reference for chemical shifts of ^1^H and ^29^Si while Al(NO_3_)_3_ × 6 H_2_O, *c*(Al(NO_3_)_3_ × 6 H_2_O) = 1 mol dm^−3^) for ^27^Al MAS NMR measurements. The spectra were deconvoluted using *dmfit* software applying Gaussian–Lorentz model.

Prior to ^1^H MAS NMR experiments, the samples nano‐sil‐1, micro‐sil‐1 and micro‐sil‐1‐F were heated at 450 °C in a vacuum chamber for 4 h (spectra in Figure 2A). Upon this treatment, the samples were exposed to water vapor, and the corresponding spectra are displayed in Figure S2B in the Supporting Information. For the spectra displayed in Figure 5, the heating of the samples at 200 °C preceded the experiments. The quantification of H sites was performed with respect to the spectrum of adamantane acquired under the same experimental settings.

The Fourier‐transformed infrared (IR) spectra of the studied materials were recorded on a Nicolet Impact 410 FTIR spectrometer equipped with a DTGS detector in the range 400–4000 cm^−1^. Pressing of the powder materials into self‐supported thin pellets preceded the measurements. The pellets were placed in the IR cell connected to the vacuum line and heated at 450 °C (2.36 °C min^−1^) for 2 h under the pressure of 10–6 torr before acquiring the spectra at room temperature. The obtained spectra were normalized to the weight of the self‐supported disc.

Thermogravimetric analysis (TG) of the samples was per‐formed by employing a Setaram Setsys TGA instrument. The samples were heated up to 800 °C with a rate of 5 °C min^−1^ in airflow. The overnight exposure of the samples to the atmosphere of 77% relative humidity preceded the TG experiments. Agilent AES 5100 VDV inductively coupled plasma atomic emission spectrometer (ICP‐AES) was employed to measure the elemental composition of the studied MFI‐type materials.

DLS analysis was used to measure the nanoparticles' hydrodynamic diameters in the suspension using a Malvern Zetasizer Nano instrument. The analyses were performed under the following conditions: scattering angle 173°, HeNe laser with 3 mW output power at 632.8 nm wavelength.

## Conflict of Interest

The authors declare no conflict of interest.

## Supporting information

Supporting Information

## Data Availability

The data that support the findings of this study are available from the corresponding author upon reasonable request.
