# Supplementary material for: Defect Sites in Zeolites: Origin and Healing
Source: Adv Sci (Weinh). 2021 Nov 27;9(4):2104414. doi: 10.1002/advs.202104414 (PMC8811801; doi:10.1002/advs.202104414)
Supplement: Supplementary file 1 — Supporting Information [file ADVS-9-2104414-s001.pdf]

WILEY-VCH

ADVANCED SCIENCE
Open Access

Supporting Information

for *Adv. Sci.*, DOI: 10.1002/advs.202104414

Defect sites in zeolites: origin and healing

Ana Palčić, Simona Moldovan, Hussein El Siblani, Aurelie Vicente, and Valentin Valtchev**

Supporting Information

Defect sites in zeolites: origin and healing

Ana Palčić, Simona Moldovan, Hussein El Siblani, Aurelie Vicente, and Valentin Valtchev**

Contents:

Supporting Figures…………………………………………………………………........S2
Supporting Table…………………………………………………………………………S6
Supporting References……………………………………………………………………S7

Supporting Figures

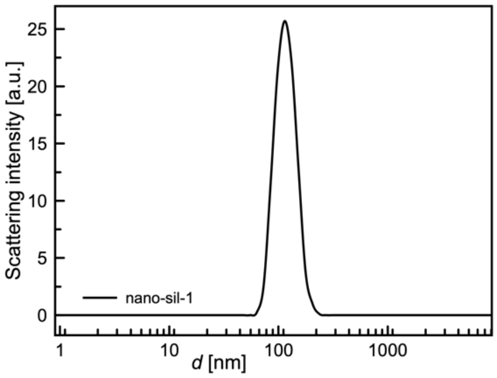

Figure S1. DLS curve of the nanosized silicalite-1 zeolite.

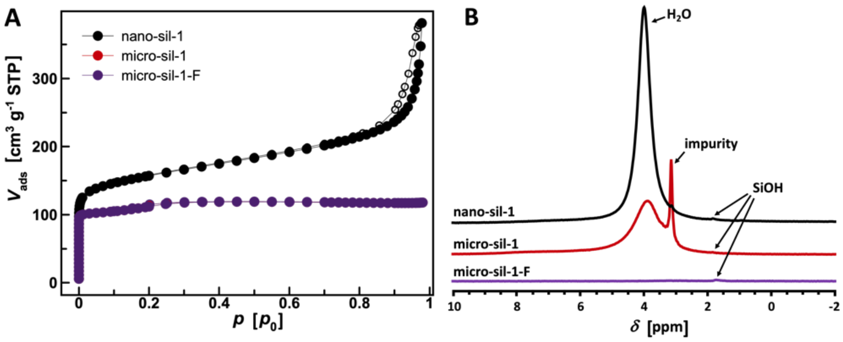

Figure S2. N_2 adsorption isotherms of the studied silicalite-1 materials (A). Closed symbols represent the adsorption, whereas open symbols the desorption branch. ^{1}H MAS NMR spectra of silicalite-1 samples prepared from different synthesis mixtures upon adsorbing water (B). The dehydration at 400 °C preceded the water adsorption.

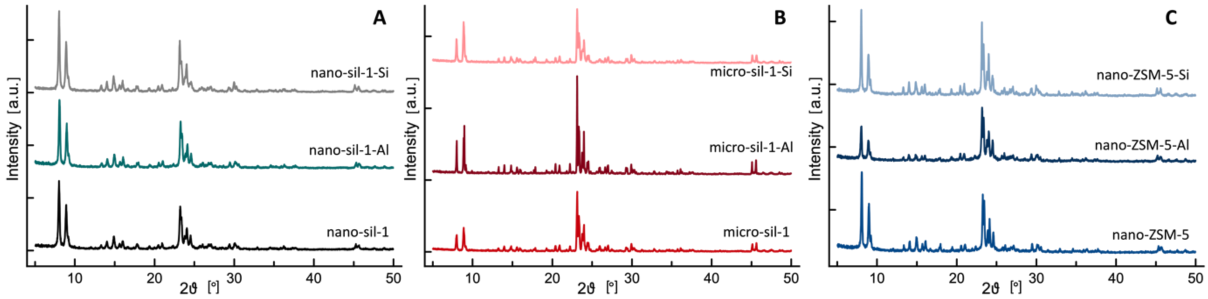

Figure S3. XRD patterns of the parent MFI-type materials (A – nano-sil-1; B – micro-sil-1; C – nano-ZSM-5) compared with the respective samples modified with Al as well as the calcined Si-treated samples.

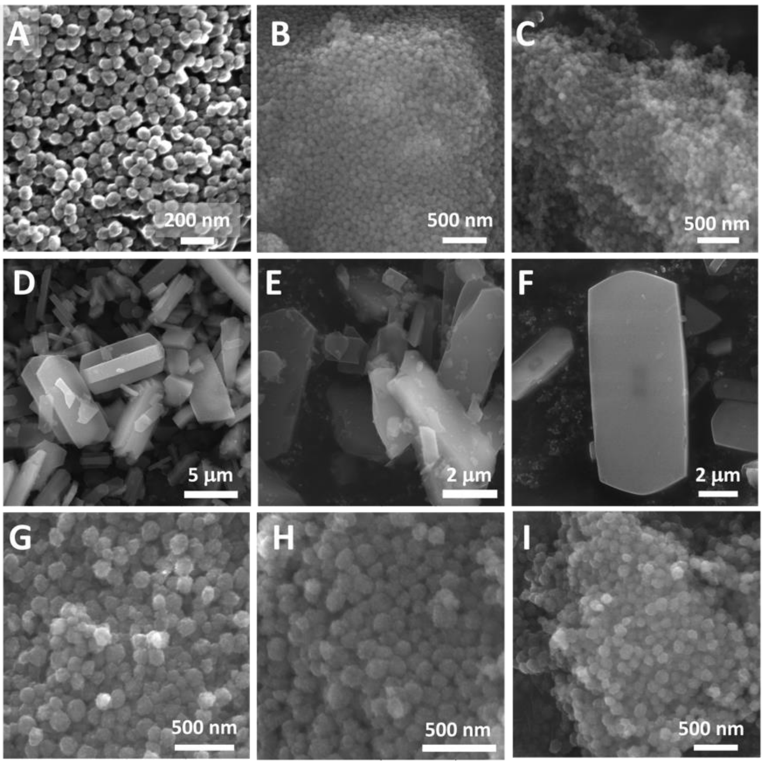

Figure S4. SEM images of the nano-sil-1 (A), nano-sil-1-Al (B), nano-sil-1-Si (C), micro-sil-1 (D), micro-sil-1-Al (E), micro-sil-1-Si (F), nano-ZSM-5 (G), nano-ZSM-5-Al (H), nano-ZSM-5-Si (I).

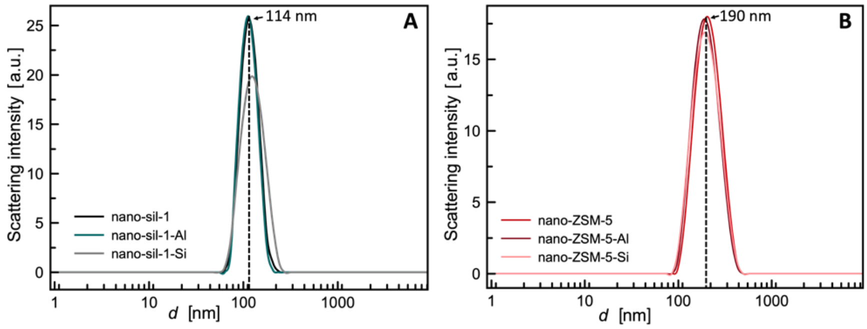

Figure S5. DLS curves of the parent nanosized MFI-type materials (A – nano-sil-1; B – nano-ZSM-5) compared with the respective samples modified with Al as well as the calcined Si-treated samples.

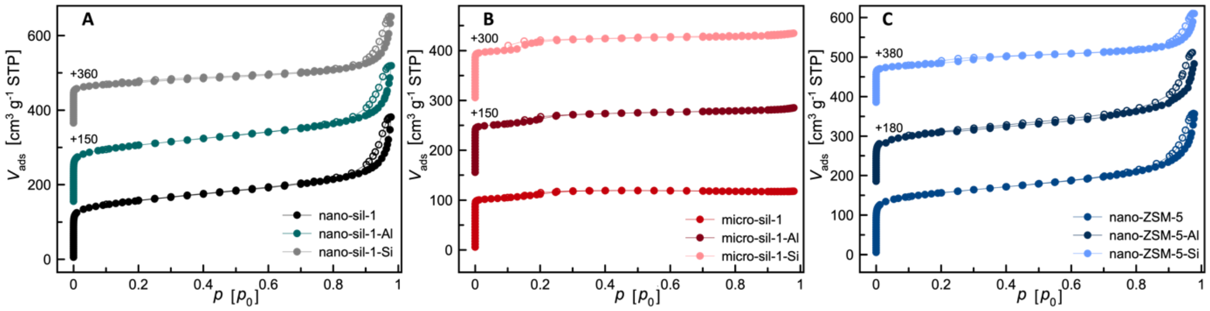

Figure S6. Nitrogen adsorption isotherms of the parent MFI-type materials compared with the respective samples modified with Al as well as the Si-treated samples (A – nano-sil-1; B – micro-sil-1; C – nano-ZSM-5). Closed symbols represent adsorption whereas open symbols correspond to desorption branch.

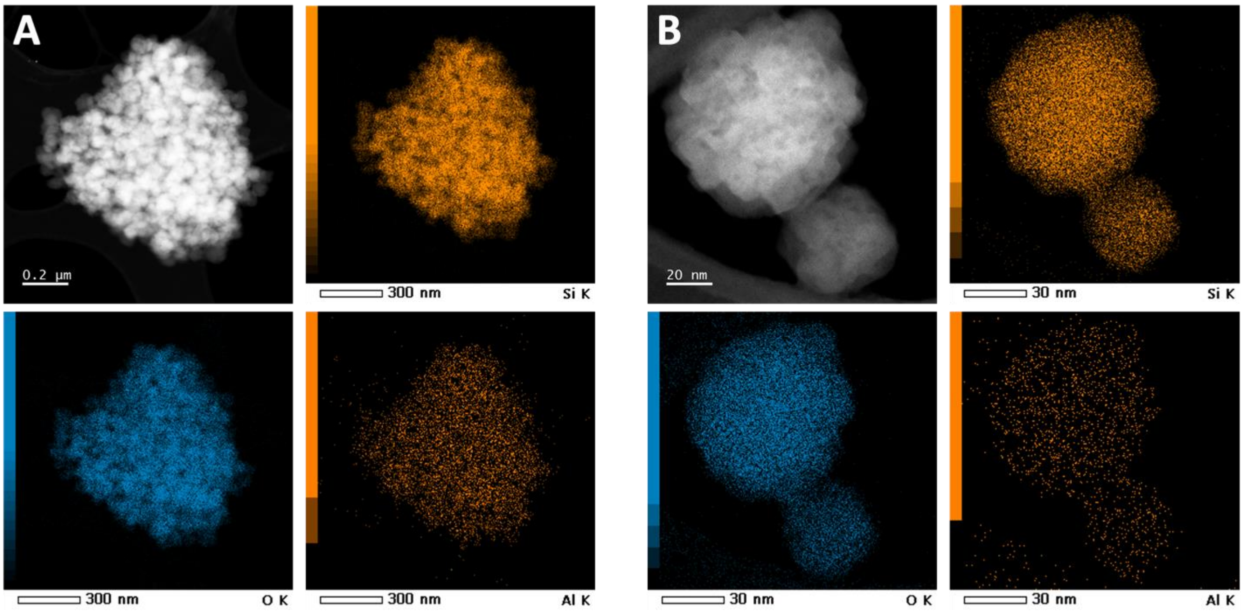

Figure S7. STEM-EDX analysis of two different regions in nano-sil-1-Al sample.

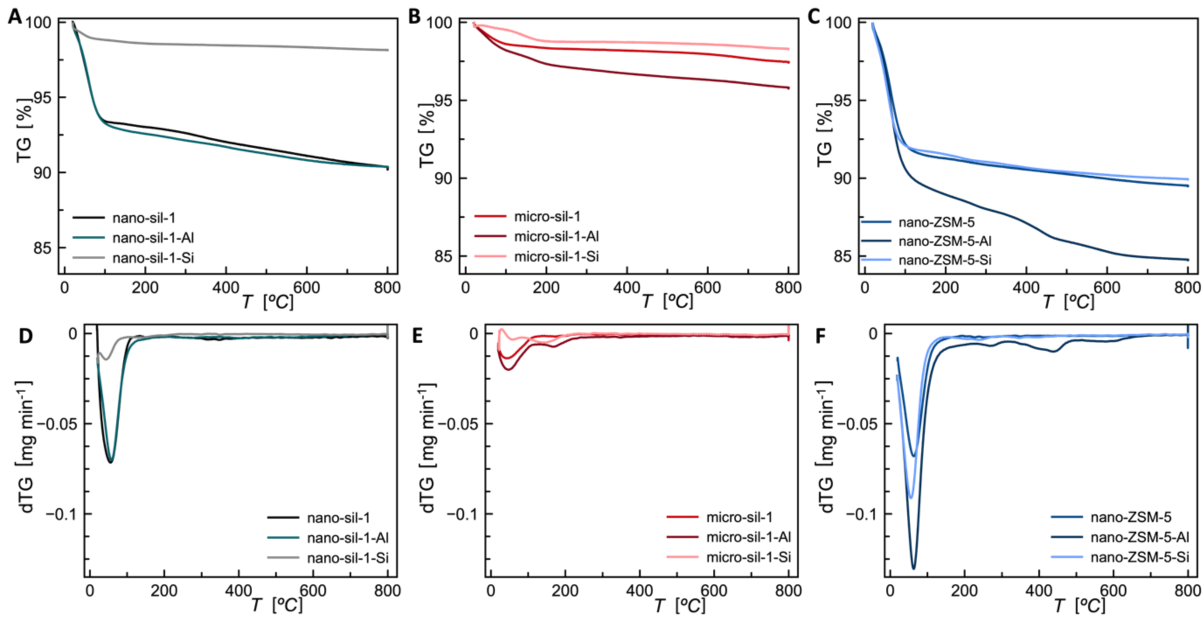

Figure S8. Thermogravimetric analysis curves and the corresponding differential thermogravimetric analysis curves of the hydrated samples from the studied series of materials: A, D – nano-sil-1; B, E – micro-sil-1; C, F – nano-ZSM-5.

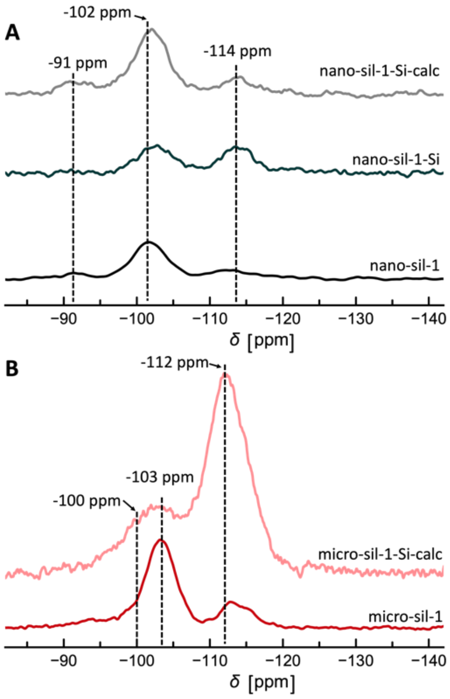

Figure S9. Normalized ^{1}H and ^{29}Si {^{1}H} cross-polarized MAS NMR spectra of the parent nanosized (A) and micron-sized (B) silicalite-1 sample and the corresponding silicated samples.

Supporting Table

Table S1. Chemical shifts of the curve maximums observed in the ^{1}H MAS NMR spectra of the studied series of samples. E.g. in the columns corresponding to micro-sil-1 are combined all signals observed in the spectra of micro-sil-1, micro-sil-1-Si and micro-sil-1-Al. The resonances were assigned on the grounds of corresponding reference.

nano-sil-1			micro-sil-1			nano-ZSM-5		
δ / ppm	assignation	Ref.	δ / ppm	assignation	Ref.	δ / ppm	assignation	Ref.
						0.8	extra-framework Al species	1
1.05	isolated silanols	2	1.05	isolated silanols	2	1.05	isolated silanols	2
1.2	isolated silanols	3	1.2	isolated silanols	3	1.2	isolated silanols	3
1.3	isolated silanols	4						
						1.4	isolated silanols	4
1.7	external silanols	5				1.7	external silanols	5
1.8	isolated internal silanols and/or geminal silanols	6,7	1.8	isolated internal silanols and/or geminal silanols	6,7			
2	internal silanols	5				2	geminal or vicinal silanol	8
			2.1	internal silanols	5			
						2.2	internal silanols	5
2.5	extra-framework AlOH	5						
						2.7	extra-framework AlOH	9
			2.8	strongly bound water	6			
						3.1	water adsorbed on dehydrated surface	10
3.2	H-bonded water	11						
			3.4	H-bonded water	11			
3.7	physically adsorbed water	12				3.7	Brønsted acid sites	5
3.9	H-bonded water	8	3.9	H-bonded water	8	3.9	H-bonded water	8
4.4	bridging SiOHAl	13,14						
			4.5	silanol nests; bridging SiOHAl	13,14	4.5	bridging SiOHAl	13,14
						5.5	bridging OH in small channels or cages	15
			5.7	liquid-like water	6			
6.7	NH_4^+ ions	16				6.7	NH_4^+ ions	16
7	disturbed bridging Si(OH)Al	3				7	disturbed bridging Si(OH)Al	3

Supporting References

[1] H. M. Kao, G. P. Grey, *J. Phys. Chem*. **1996**, *100*, 5105.

[2] I. S. Protsak, Y. M. Morozov, W. Dong, Z. Le, D. Zhang, I. M. Henderson, *Nanoscale Res. Lett*. **2019**, *14*, 160.

[3] M. Hunger, *Catal. Rev. Sci. Eng*. **1997**, *39*, 345.

[4] M. Trzpit, S. Rigolet, J.-L. Paillaud, C. Marichal, M. Soulard, J. Patarin, *J. Phys. Chem. B* **2008**, *112*, 7257.

[5] Z. Qin, L. Lakiss, L. Tosheva, J.-P. Gilson, A. Vicente, C. Fernandez, V. Valtchev, *Adv. Funct. Mater.* **2014**, *24*, 257.

[6] J.-B. d'espinose de la Caillerie, M. R. Aimeur, Y. l Kortobi, A. P. Legrand, *J. Coll. Interf. Sci.* **1997**, *194*, 434.

[7] C. E. Bronnimann, I.-S. Chuang, B. L. Hawkins, G. E. Maciel, *J. Am. Chem. Soc*. **1987**, *109*, 1562.

[8] T. Karbowiak, M.-A. Saada, S. Rigolet, A. Ballandras, G. Weber, I. Bezverkhyy, M. Soulard, J. Patarin, J.-P Bellat, *Phys. Chem. Chem. Phys*. **2010**, *12*, 11454.

[9] J. R. Sohn, S. J. DeCanio, P. O. Fritz, J. H. Lunsford, *J. Phys. Chem.* **1986**, *90*, 4847.

[10] V. V. Turov, S. Chodorowski, R. Leboda, J. Skubiszewska-Zięba, V. V. Brei, *Colloid. Surf. A*. **1999**, *158*, 363.

[11] J. Van Aelst, M. Haouas, E. Gobechiya, K. Houthoofd, A. Philippaerts, S. P. Sree, C. E. A. Kirschhock, P. A. Jacobs, J. A. Martens, B. F. Sels, F. Taulelle, *J. Phys. Chem. C* **2014**, *118*, 22573.

[12] G. Qi, Q. Wang, J. Xu, Q. Wu, C. Wang, X. Zhao, X. Meng, F. Xiao, F. Deng, *Commun. Chem.* **2018**, *1*, 22.

[13] M. Hunger, S. Ernst, S. Steuernagel, J. Weitkamp, *Micropor. Mater*. **1996**, *6*, 349.

[14] C. C. Liu, G. E. Maciel, *J. Am. Chem. Soc*. **1996**, *118*, 5103.

[15] D. Freude, J. Kärger, in *Handbook of Porous Solids*, vol. 1. (Eds.: F. Schüth, K. S. W. Sing, J. Weitkamp) Wiley-VCH, Weinheim, 2002, Ch. 2.12.

[16] C. Doremieux-Morin, J. Fraissard, *Sekiyu Gakkaishi*, **1997**, *40,* 355.
